# Biosecurity messages are lost in translation to citizens: Implications for devolving management to citizens

**DOI:** 10.1371/journal.pone.0175439

**Published:** 2017-04-12

**Authors:** Marnie L. Campbell, Dominic E. P. Bryant, Chad L. Hewitt

**Affiliations:** 1 University of Tasmania, National Centre for Marine Conservation and Resource Sustainability, Locked Bag 1370, Newnham, Tasmania, Australia; 2 Environmental Research Institute, University of Waikato, Hamilton, New Zealand; 3 School of Science, University of Waikato, Hamilton, New Zealand; Smithsonian Environmental Research Center, UNITED STATES

## Abstract

The increasing focus of marine biosecurity agencies on transferring management responsibilities to citizens and industry begs the question whether devolved responsibility is a viable option for creating biosecurity outcomes. We examined recreational marine users’ self-declared awareness of non-indigenous marine species (NIMS) at six locations in Tasmania, Australia and evaluated the accuracy of their awareness through recognition of four well-known NIMS with active awareness campaigns. We also investigated whether the activities of recreational marine users influence the accuracy of their NIMS recognition skills. We generally found that respondents declare NIMS awareness (70.45%), yet we found their recognition accuracy was variable ranging from low to fair (<10% to 54.95%) and recreational activity did not influence accuracy. Based on our results, we conclude that marine users’ awareness does not predict accuracy and therefore devolved management of biosecurity without additional resources may pose a risky biosecurity management strategy.

## Introduction

The management of non-indigenous marine species (NIMS) is increasingly reliant on contributions from the lay public through ‘citizen science’ and self-regulation. Citizens can contribute to the management of NIMS by detecting and reporting “pests”, helping with eradication (e.g., [1–4]), and aiding in vector management, such as self-managing their equipment and vessels (e.g., Marine Biosecurity–Vessel Cleaning [5]). In some jurisdictions (e.g., Australia and New Zealand) the concept of a ‘biosecurity citizen’ exists and is ensconced in the regulatory stance and legislation [6, 7]. In New Zealand, biosecurity citizens have contractual and non-contractual responsibilities to participate in detection, surveillance and reporting of non-indigenous species [7]. This increasing reliance on citizens represents a movement towards the devolution of biosecurity by the state to NIMS vector operators, end users and stakeholders.

The devolution of management, particularly in border control and quarantine, may extend to self-regulation where the government grants devolved responsibility to manage biosecurity controls, despite inherent conflicts of interest that may arise. For example, some Australian aquarium fish importers have delegated approvals to act as border controlled facilities and providing quarantine at their own facilities, often acting both as wholesaler and direct retailer (personal observations). The success of this devolution relies on a shared commitment to, and understanding of, biosecurity ideals (e.g., [7]) and the capability to recognise diseases, parasites and pests associated with imports.

Participatory management provides a sense of democratic process that is responsive, inclusive (of public and indigenous knowledge) and transparent, enabling public scrutiny of management agencies and decisions [7, 8]. Devolution of management potentially reduces operating costs (e.g., [1, 9]), theoretically freeing up funds to be spent on enhancing scientific knowledge and response interventions, and it also empowers the community and potentially increases compliance, similar to protected area management enforcement [10]. However a move towards self-governance also has trade-offs, such as limited compliance control or enforcement from the management agency [10] and more uncertainty concerning data quality [3, 11].

Quarantine aims to prevent a non-indigenous species from crossing a border, but the management aim switches from protecting the border to preventing or minimising the impacts of NIMS within country once the border is breached [12–14]. The focal shift from pre- to post-border puts an onus on citizens to be aware of, and willing to engage with, biosecurity ideals. A number of benefits from including citizens are anticipated including increased buy-in and a wider and more frequent geographic coverage providing early warning of new incursions and spread (e.g., [6, 7, 15]).

Effective citizen education and outreach programs are needed if devolved self-management and self-regulation are to prosper and succeed. For marine biosecurity, this is often addressed by creating citizen awareness of NIMS, their impacts, and management programs, which may involve encouraging citizens to provide information of a suspected NIMS (i.e., pest hotlines, web reporting) (e.g., [16]). Intrinsic to these public education and outreach programs is the desire to shift individual behaviours to ones that support management prerogatives. By doing so, this provides a sense of empowerment to citizens by raising their awareness and providing mechanisms for them to help make responsible judgements regarding their actions. The effectiveness of such systems is difficult to gauge as “no detection of a pest” may result for a number of possible reasons including: 1) the pest has not arrived and therefore cannot be detected; 2) no one is looking for the pest; or 3) a failure to recognise the pest arrival (e.g., incursion). The latter represents a Type II error where a NIMS is mis-identified as a native, and where devolved responsibility relies on detection and recognition of a pest (the “biosecurity message”) this Type II error represents a failure of biosecurity protections. So how frequently is the “biosecurity message” lost in translation?

To explore this concept, we investigated self-rated (i.e., declared) recreational marine users’ ability to recognise and differentiate four well-established NIMS from similar natives (i.e., accuracy) in Tasmania. We also explored the influence of recreational marine user activity on recognition accuracy to determine if different marine user groups could effectively contribute to detection and reporting of NIMS. This work aimed to challenge the efficacy of self-regulation of biosecurity in a marine context using Tasmania, Australia, as a case study.

## Materials and methods

### Ethical statement

This research was conducted in strict accordance with the [Australian] National Statement on Ethical Conduct in Research Involving Humans. The research protocol was approved by the University of Tasmania Human Research Ethics Committee, project number H10546. Prior to completing the questionnaire, respondents were provided an introduction to the activity and asked to signify willingness to participate (i.e., provide informed consent) and to confirm that they were over 18 years of age.

### Survey methods

**Fig 1:**
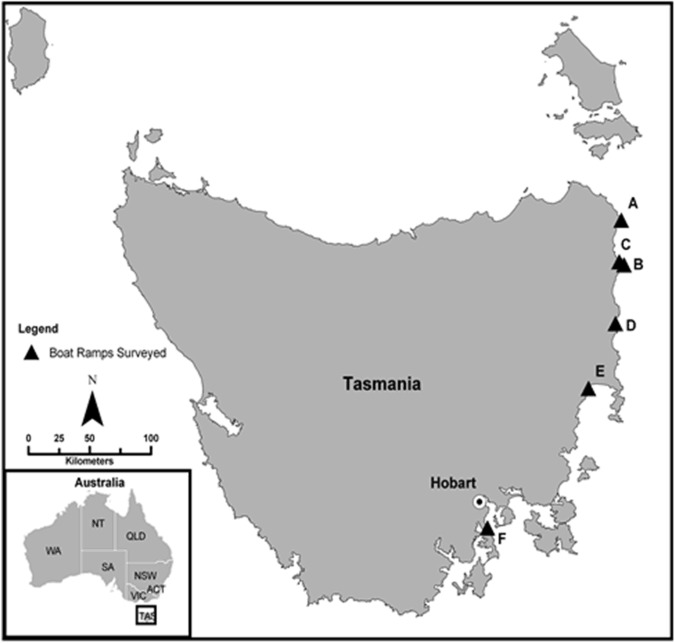
Map of the six boat ramps used for surveys on the east coast of Tasmania. Where A = Eddystone point (-40°59’ 24.07 S, 148°20’44.41 E), B = Binnalong Bay (-41°20’23.51”S, 148°16’15.39”E), C = St Helens (-41°16’ 37.40”S, 148°20’55.96”E), D = Bicheno (-41°55’28.07”S, 148°18’14.04”E), E = Triabunna (-42°30’18.33”S, 147°54’28.97”E), F = Tinderbox (-43°03’31.93”S, 147°19’48.68”E).

A questionnaire survey was developed and implemented during four holiday periods (April 2009, December 2009, January 2010 and April 2010) via face-to-face interviews at six boat ramps on the east coast of Tasmania in close proximity to marine protected areas (Fig 1). The Tasmanian State government provides NIMS awareness signage at all primary boat ramps that includes photos of key established NIMS. In addition, awareness pamphlets are provided with all boat licenses, providing the opportunity for NIMS awareness saturation in marine recreational users. We undertook interviews during holiday periods and locations close to Marine Protected Areas (MPAs) were targeted to increase the likelihood of obtaining a statistically robust sample size, as these areas often attract tourists and are associated with recreational marine activities.

The questionnaire consisted of 45 questions and took respondents approximately 10–15 minutes to complete. Surveyors targeted people that were seen to be participating in or possessing equipment needed for obvious recreational marine activities (e.g., boating, fishing, kayaking, and SCUBA diving) close to the selected boat ramps (our geographical anchor points). We employed a stratified convenience method, targeting every second person within the vicinity of the boat ramp. The questionnaire components relevant to this paper (see S1 Appendix) were divided into three focal areas:

1. Demographics (independent variables): postcode (surrogate for proximity to site), age, gender, income, and education level (see [17]);
2. Marine activities and behaviours (independent variables): users were asked to identify all marine activities that they participated in on the day, and which marine activities they participate in in general. Marine activities were further categorised into three types of sub-activity for analyses: a) whether the activity was based on the surface, or below the water (underwater); b) if the activity intent was observational or capture and/or collection of wildlife; and c) for capture/collection activities, whether the user required a license to capture/collect organisms, or if the activity involved non-licensed capture/collection of organisms. All respondents were recreational users of the marine environment, with commercial users being excluded; and
3. Accuracy of NIMS recognition (dependent variables): The Tasmanian recreational marine user participants were asked a binary (yes/no) question to determine if they are aware of NIMS in Tasmania. To determine if their declared awareness was accurate (if they proclaimed to be aware, could they recognise NIMS already present in Tasmania), respondents were asked to identify the correct image, amongst three images, for each of four common NIMS that are declared as “pests”: *Undaria pinnatifida* (Harvey) Suringar, 1873 (wakame, the Japanese kelp), *Asterias amurensis* Lutken, 1871 (northern Pacific seastar), *Maoricolpus roseus* (Quoy & Gaiman, 1834) (New Zealand screwshell) and *Carcinus maenas* (Linnaeus, 1758) (European green crab). The NIMS images used are provided in S1 Appendix.

### Data analysis

#### Overall awareness and recognition accuracy

The overall declared awareness of NIMS for all respondents was determined by comparing the percentage of “aware” and “unaware” respondents against their level of accuracy when recognising NIMS. The number of respondents who answered correctly, misidentified one species for another, or decided they did not know, was recorded and Chi-squared (χ^2^) tests of independence were performed to determine if there was a statistically significant difference between “aware” and “unaware” respondents’ ability to identify NIMS, to determine if any one species was more accurately identified than others, and to test for relationships between different marine user groups and declared awareness, using an alpha of 0.05 to determine significance. Similarly, the accuracy of respondents was also assessed by evaluating the number of correctly identified species.

## Results

**Fig 2:**
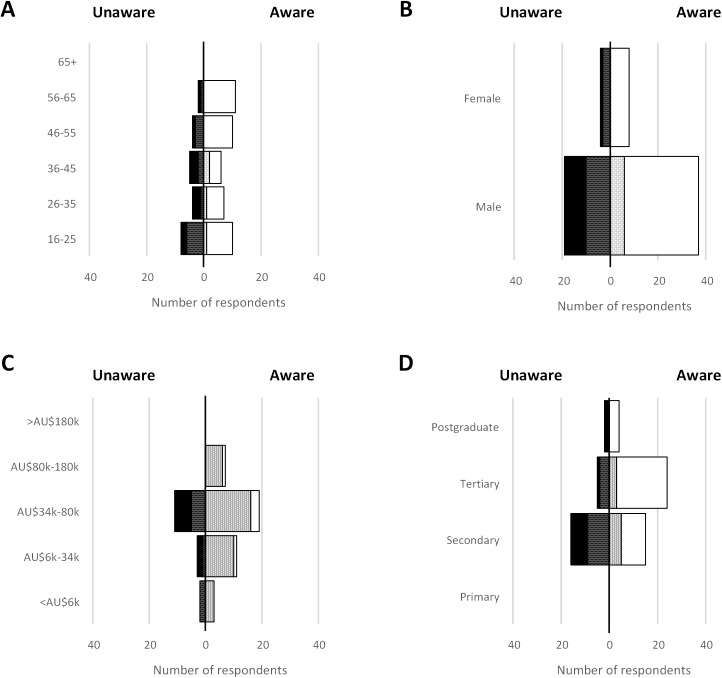
Survey respondent distribution for A) Age, B) Gender, C) Income; and D) Education with self-declared “unaware” respondents to the left (black and dark stipple) and “aware” respondents to the right (white and light stipple). Solid (black and white) represents accurate recognition of at least two NIMS; stippled (dark stipple and light stipple) represents accurate recognition of less than two NIMS.

**Fig 3:**
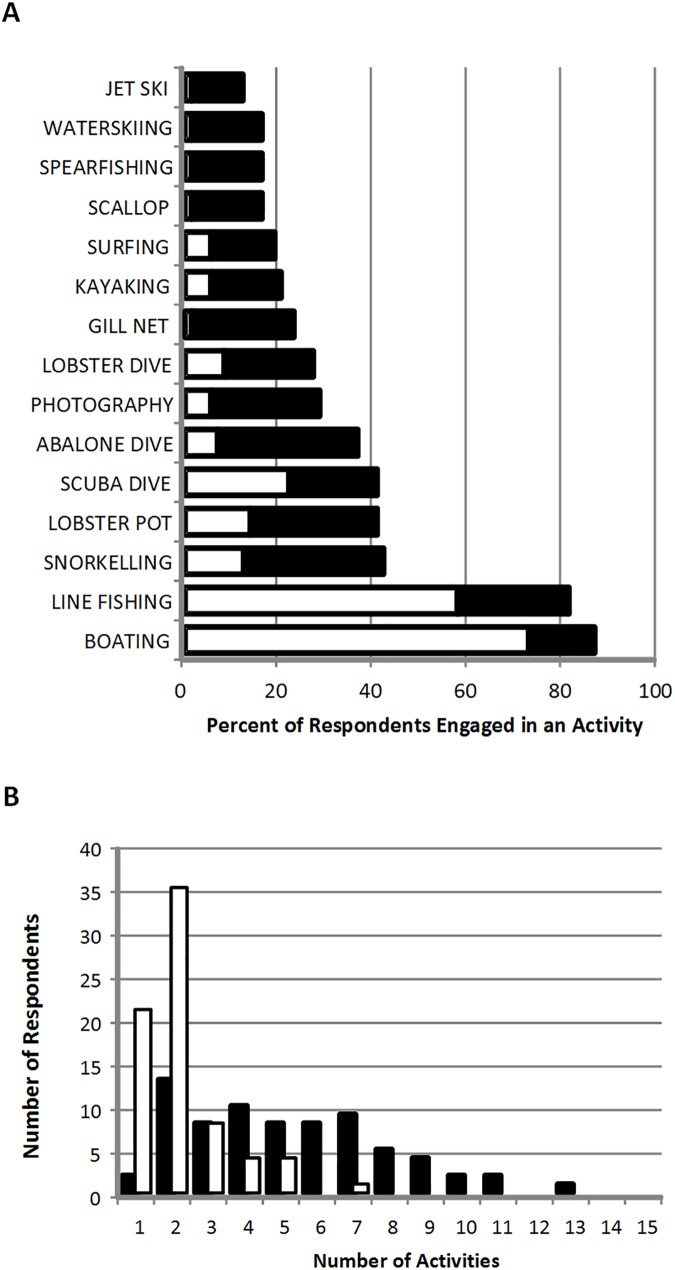
Survey respondents’ participation in marine activities A) on the day of survey and in general, and B) the number of cumulative activities for individual respondents. Black bars represent general participation in an activity and white bars represent participating in that activity on the day approached. Note that marine activities are not mutually exclusive for a respondent.

Overall 137 respondents completed the questionnaires, which was representative of our Tasmanian beach users sample frame. The demographic profile highlights that respondents were spread across age groups between 16 and 65, and the majority were male (82.3%), with secondary or tertiary education (90.9%), earning AU$34k-80k (53.6%) (Fig 2; S1 Table). We note that the gender gap among respondents is striking, but is a true reflection of the population of marine users at our sampling sites and during the sampling period [17]. On the day of the survey, and in general, the majority of respondents undertook boating and line fishing (Fig 3A). A large proportion of respondents undertook a small number of activities on any given day, but undertook multiple marine activities in general (Fig 3B).

### Overall awareness and recognition accuracy

**Fig 4:**
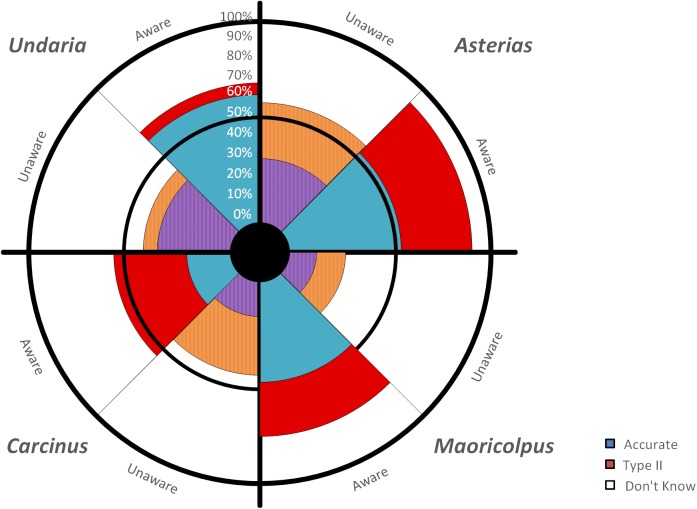
Respondent percentage of responses (accurate–blue; misidentification (Type II error)–red; “don’t know”—white) for each of the four selected NIMS (*Undaria pinnatifida*, *Asterias amurensis*, *Maoricolpus roseus* and *Carcinus maenas*); for “Aware” and “Unaware” groups.

The majority (70.45%) of respondents believed that they were aware of NIMS in Tasmania. There was a significant relationship between level of recognition accuracy across the four NIMS (χ^2^_[3]_ = 58.93, P<0.001; Fig 4). Overall (regardless of self-declared awareness), respondents had a 38.7% recognition accuracy, 23.9% misidentification (Type II error) and 37.4% “don’t know”. The most recognisable species was *Undaria pinnatifida*, with 45.6% of respondents that attempted to identify the species being correct. Successful recognition of *Asterias amurensis* was low (39%), with this species being commonly misidentified (41.2%) as either *Astrostole scabra* (also a non-indigenous seastar) or *Fromia polypore* (a native seastar). *Carcinus maenas* was the least accurately recognised species, with an accuracy of 22.1% (Fig 4).

Declared awareness was significantly related to accuracy levels for *U*. *pinnatifida* (χ^2^_[2]_ = 12.53, P>0.001), *A*. *amurensis* (χ^2^_[2]_ = 13.94, P<0.001), *M*. *roseus* (χ^2^_[2]_ = 11.73, P<0.001), but not for *C*. *maenas* (χ^2^_[2]_ = 2.64, P>0.05). A majority of aware respondents correctly recognised *U*. *pinnatifida* (62.8%), *A*. *amurensis* (55.8%), *M*. *roseus* (54.2%), however only 20.9% of aware respondents correctly recognised *C*. *maenas* (Fig 4). Unaware respondents exhibited moderate levels of accuracy for *U*. *pinnatifida* (35.7%) and *A*. *amurensis* (32.1%), but low levels for *M*. *roseus* (14.3%) and *C*. *maenas* (17.9%) (Fig 4).

Self-declared aware respondents were 1.2 to 2.7 times more likely to attempt to identify a species than unaware respondents. Misidentification (Type II error) was low for *U*. *pinnatifida* for both aware (7.0%) and unaware (7.1%) respondents. For all other species the levels of misidentification were between 14% and 37.2%, with higher proportions of aware respondents misidentifying species than unaware respondents (Fig 4). It is noteworthy that unaware respondents were more likely to choose the “don’t know” option than aware respondents for all four NIMS (Fig 4).

**Fig 5:**
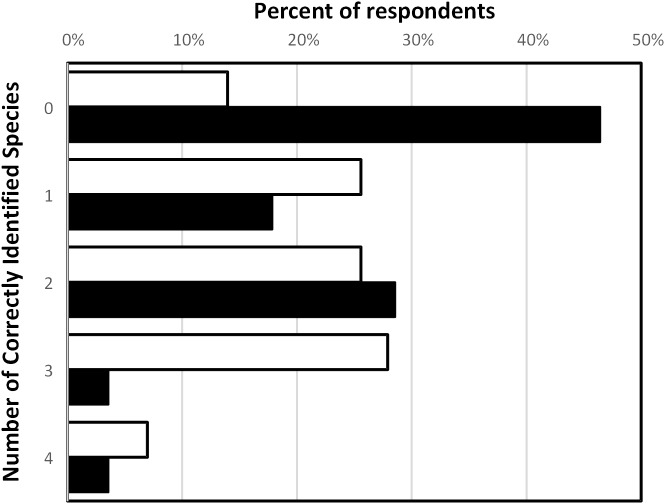
Accuracy levels (as number of correctly identified species) of recreational marine users at recognising NIMS based on declared awareness. Black bars represent ‘unaware’ respondents and white bars represent ‘aware’ respondents.

The accuracy of species recognition was low for self-declared “unaware” individuals. Self-declared “aware” individuals had a slightly higher accuracy with an average of two accurate NIMS recognitions (Fig 5). Only four individuals were able to accurately recognise all four NIMS (three declared as being “aware”), and 34.9% (15 “aware” individuals) were able to accurately recognise three or four NIMS. In contrast, only two “unaware” individuals (7.1%) accurately identified more than three NIMS. Six “aware” individuals (13.9%) and 13 “unaware” individuals (46.4%) were unable to accurately recognise any of the four NIMS.

### Awareness and recognition accuracy levels for different user groups

#### Demographics

Males tended to declare that they were aware more frequently, but females that were aware, were 100% accurate when they attempted identification. A respondents self-reported level of education attained was positively related to the proportion of respondents claiming ‘awareness’–a higher percentage of secondary educated respondents self-identified as unaware, shifting to a higher percentage of tertiary and postgraduate educated respondents self-identified as aware, with a similar increase in accuracy of identification (Fig 2).

#### Marine activity

Awareness of NIMS in Tasmania was not significantly related to users being exclusively above water, or participating in underwater activities (χ^2^_[1]_ = 2.010, P>0.05). Furthermore, there was no significant relationship between above and underwater marine users for accuracy levels when identifying each NIMS; *U*. *pinnatifida* (χ^2^_[2]_ = 2.34, P>0.05), *A*. *amurensis* (χ^2^_[2]_ = 1.550, P>0.05), *M*. *roseus* (χ^2^_[2]_ = 0.0173, P>0.05) and *C*. *maenas* (χ^2^_[2]_ = 0.763, P>0.05).

Declared awareness was not statistically related to whether a respondent was an ‘observational’ marine user or a ‘capture’ marine user (χ^2^_[1]_ = 0.0702, P>0.05). There was also no relationship between these user groups and accuracy levels when identifying each NIMS; *U*. *pinnatifida* (χ^2^_[2]_ = 1.838, P>0.05), *A*. *amurensis* (χ^2^_[2]_ = 0.323, P>0.05), *M*. *roseus* (χ^2^_[2]_ = 2.634, P>0.05) and *C*. *maenas* (χ^2^_[2]_ = 1.286, P>0.05).

Taking part in licensed or non-licensed fishing activities was not statistically related to declared awareness of NIMS (χ^2^_[2]_ = 1.036, P>0.05) despite distribution of NIMS awareness materials to licensed individuals. There was also no relationship between participating in a licensed activity or non-licensed activity and accuracy levels when identifying the four NIMS; *U*. *pinnatifida* (χ^2^_[4]_ = 1.519, P>0.05), *A*. *amurensis* (χ^2^_[4]_ = 1.267, P>0.05), *M*. *roseus* (χ^2^_[4]_ = 3.548, P>0.05) and *C*. *maenas* (χ^2^_[4]_ = 3.596, P>0.05).

## Discussion

The delivery of biosecurity outcomes in the marine environment is difficult and costly [12, 18, 19]. The need for a diffuse observation network to enhance early detection and rapid response has encouraged use of citizen and industry engagement to supplement state based efforts (e.g., [4, 20–22]. The early successes associated with citizen based contributions have encouraged some jurisdictions to devolve biosecurity responsibilities through self-regulation and self-management, in some cases replacing state based efforts. The ability of citizens to deliver biosecurity outcomes critically relies on the adherence to and understanding of the biosecurity messages, including the ability to accurately recognise and act on pests. Our results demonstrate that although 70.4% of respondents believe that they are familiar with and aware of NIMS, their actual NIMS recognition accuracy is poor. Fewer than 50% of attempts to recognise any one species of NIMS are correct, averaging 38.7% accuracy for all four species (Fig 4). The outcome is that these errors of mis-identification potentially compromise biosecurity outcomes.

Marine bioinvasion research has typically considered biological and ecological phenomena [12, 18, 23–24]; with little consideration of the human dimensions that drive invasion outcomes and management. Awareness of human behaviours that cause invasion, NIMS identification, and the ability of citizens to harness their knowledge toward strong biosecurity outcomes has not been critically evaluated until now. These social dimensions are critical to the increasing desire for devolving biosecurity management to citizens and industry (e.g., [4, 7, 20, 25]). The current mechanisms to involve the public and citizen science in biosecurity frameworks provide potentially powerful public relations tools (e.g., [4, 8, 20]). The questions remains whether citizens are sufficiently aware and accurate (can they correctly recognise and identify NIMS), to devolve biosecurity obligations? Although mentioned in some publications (see [26–28]), we note that there are few examples of efforts focussed on determining the efficacy of biosecurity public relation exercises (e.g., [29]), with no reported rigorous evaluation of pre- and post-campaign marine biosecurity exercises in the published literature. To the best of our knowledge, the effectiveness of a devolved marine biosecurity strategy has never been examined or published.

Respondents appear to have been exposed to biosecurity messages through education campaigns and material creating a sense of NIMS awareness, but appear not to have assimilated this information [17]. We interpret this finding to mean that these biosecurity messages have been lost in translation. The role of many public education and outreach systems, including NIMS programs, are typically focused on influencing people’s capabilities and behaviours (e.g., [30]). The general reasoning is that if individuals become aware of NIMS and their impacts, and are empowered to take action, they may be co-opted into being biosecure citizens and promptly notify authorities to enable a rapid and successful response (e.g., [4]).

While the fiscal benefits of devolved biosecurity management are desirable, the concern is that biosecure citizens may remain unaware of NIMS or have faulty (inaccurate) knowledge. By extension, individuals would potentially mis-manage their own behaviours to reduce the likelihood of moving NIMS (such as managing vessel biofouling). We recognise that state-based biosecurity approaches to vector management provide broad benefit, and may require additional consideration of reliance on self-management. Our results indicate that this expectation of creating biosecure citizens may create a false sense of security for managers who perceive a higher-than-realistic level of community capability. The knock-on effect could lead to biosecurity managers being over-confident in their devolved strategy, resulting in poor management behaviours and choices, with mis-allocation of resources.

The devolution of management to end-users has some similarities to citizen science activities used to collect and bolster scientific data. Effective and successful citizen science efforts are well-documented (e.g., [4, 8]), including partnerships between citizens and environmental managers that aim to increase learning outcomes [31], provide empowerment, and increase local capacity (e.g., [32]). Yet recognised issues exist in citizen science. A common criticism is data quality and reliability (e.g., [8, 33]), where reliable data are a critical foundation for successful environmental management [34].

Riesch & Potter [8] suggest improving citizen science data quality by ensuring appropriate training and/or close supervision, simplifying tasks for non-scientists, and validation. Doing so has a fiscal cost. Delaney et al. [3] note that citizens can accurately collect data on native and non-indigenous crabs, but only after receiving adequate training to identify target species. Similarly, Finn et al. [35] highlighted that accurate citizen collection of seagrass cover required training in simple methods. Science-education campaigns have improved the use of citizens to collect information, but based on our findings we question whether citizen scientists are sufficiently accurate and precise to justify self-regulation when Type II errors have such potentially high costs.

This study has clearly shown that the respondents are overconfident (i.e., dispositional optimism or mis-calibration [36, 37]) in their abilities to accurately identify the test species. We did not investigate the source of this overconfidence, but recommend that investigation is needed. A respondent that believes that they are aware of NIMS, but cannot recognise them, creates a false sense of a biosecure plebiscite and in turn results in overconfidence in managers (personal observations). If management assumes a precautionary approach (e.g., [38–40]) then inaccurate knowledge represents a hazard that needs to be managed.

Inaccurate knowledge will increase mis-identifications and therefore increase both Type I and Type II errors. In this case, Type I errors represent false identification and reporting of NIMS and are likely to result in increased expenditure due to responses to natives falsely identified as pests. In contrast, Type II errors represent false identification of NIMS as natives resulting in no reporting and therefore no management action–a significant biosecurity risk. Unfortunately, this study demonstrates that individuals who deem themselves to be “aware” of NIMS have high levels of inaccuracy (23.9% on average) or don’t attempt identification (37.3%). Less than 40% of respondents were able to accurately recognise more than three of the four NIMS, suggesting that high levels of Type II errors will accrue. The identification of well-trained biosecure citizens will be more difficult than previously anticipated. Thus, the trade-off between engaging the community as a public relations exercise and using biosecure citizenry as an element of frontline management needs to consider the implications to both biosecurity outcomes and budgets.

## Supporting information

S1 Table Demographic profile of respondents from six boat ramps in Tasmania. Numbers in brackets represent percentage of overall respondents.
(DOCX)

S1 Appendix Survey Questions. (DOCX)
