# Supplementary material for: Biosecurity messages are lost in translation to citizens: Implications for devolving management to citizens
Source: PLoS One. 2017 Apr 12;12(4):e0175439. doi: 10.1371/journal.pone.0175439 (PMC5389827; doi:10.1371/journal.pone.0175439)
Supplement: S1 Table — Numbers in brackets represent percentage of overall respondents. (DOCX) [file pone.0175439.s001.docx]

**SI Table.** Demographic profile of respondents from six boat ramps in Tasmania. Numbers in brackets represent percentage of overall respondents

| ***N*=74** |  | **Total (%)** |
| --- | --- | --- |
|  |  |  |
| Age | 16-25 | 19 (26) |
|  | 26-35 | 13 (18) |
|  | 36-45 | 13 (18) |
|  | 46-55 | 15 (20) |
|  | 56-65 | 13 (18) |
|  | 66+ | 1 (1) |
|  |  |  |
| Sex | Male | 61 (82) |
|  | Female | 13 (18) |
|  |  |  |
| Income | <AU$6k | 5 (7) |
|  | AU$6k-34k | 15 (20) |
|  | AU$34k-80k | 29 (39) |
|  | AU$80k-180k | 9 (12) |
|  | >AU$180,000 | 0 (0) |
|  | Not Answered | 12 (16) |
|  |  |  |
| Education level | Primary | 1 (1) |
|  | Secondary | 32 (43) |
|  | Tertiary | 30 (41) |
|  | Postgraduate | 9 (12) |
|  | Not Answered | 2 (3) |
