# Supplementary material for: Biosecurity messages are lost in translation to citizens: Implications for devolving management to citizens
Source: PLoS One. 2017 Apr 12;12(4):e0175439. doi: 10.1371/journal.pone.0175439 (PMC5389827; doi:10.1371/journal.pone.0175439)
Supplement: S1 Appendix — (DOCX) [file pone.0175439.s002.docx]

**SI Appendix**

**Survey Questions**

Please select which boat ramp you visited today:

- Tinderbox
- Triabunna
- Bicheno
- St Helens
- Mt. Williamson National Park (Eddystone point)
- Other

**Section 1. Demographics**

***These* *few questions are required to analyse the results of this survey by comparing your responses with others. All answers are kept completely confidential***

1. What is you postcode?
2. What is your age? (Circle one)
   - 16-25
   - 26-35
   - 36-45
   - 46-55
   - 56-65
   - 65 and over
3. What is your gender? (Circle one)
   - Male
   - Female
   - Choose not to say
4. What is your yearly income (Circle one)?
   - AU$1-6000
   - AU$6001-34000
   - AU$34001-80000
   - AU$80001-180,000
   - AU$1800000+
5. What is you highest level of education?
   - Primary
   - Secondary
   - Tertiary
   - Postgraduate

**Section 2. Activities undertaken**

1. Please tick all marine activities you participate in.
   - Boating
   - Line & rod fishing
   - Spearfishing
   - Gill net fishing
   - Rock lobster & crab pot fishing
   - Scuba diving
   - Snorkelling
   - Photography
   - Abalone fishing
   - Rock lobster (diving)
   - Kayaking
   - Surfing
   - Waterskiing
   - Jet ski
   - Scallop fishing
   - Other?
2. Which activities are you planning on DOING TODAY (please select all that are relevant)
   - Boating
   - Line & rod fishing
   - Spearfishing
   - Gill net fishing
   - Rock lobster & crab pot fishing
   - Scuba diving
   - Snorkelling
   - Photography
   - Abalone fishing
   - Rock lobster (diving)
   - Kayaking
   - Surfing
   - Waterskiing
   - Jet ski
   - Scallop fishing
   - Other
3. How often do you come to this boat ramp for marine activities in a year?
   - Once a week
   - Once a month
   - Once every six months
   - Once a year
   - Other
4. How often do you undertake any marine activities a year?
   - Once a week
   - Once a month
   - Once every six months
   - Once a year
   - Other

**Section 3. Invasive species awareness**

1. Are you aware of introduced marine species in Tasmania?
   - Yes
   - No
   - Unsure

**For the following images can you please select a picture that you feel is accurate?**

1. Which of the following pictures do you think is Japanese kelp “wakame” (*Undaria pinnatifida*)?
   - [
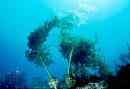
](http://images.google.com/imgres?imgurl=http://www.marine.csiro.au/crimp/images/NIMPIS/Undaria_pinnatifida.jpg&imgrefurl=http://www.marine.csiro.au/crimp/nimpis/spSummary.asp?txa=6808&usg=__Q9-d-VNtF9otj-Xe0yBCLX5V-4A=&h=344&w=500&sz=84&hl=en&start=3&um=1&tbnid=rlOyeCzJTu1_DM:&tbnh=89&tbnw=130&prev=/images?q=undaria+pinnatifida&hl=en&rls=com.microsoft:*:IE-SearchBox&rlz=1I7GGIK_en&sa=N&um=1)
   -
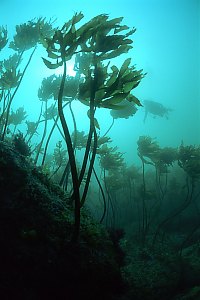

   -
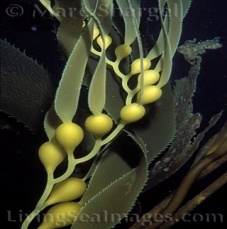

   - Don’t know
2. Which of the following pictures do you think is the Northern Pacific sea star (*Asterias amurensis*)?
   -
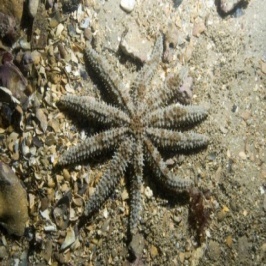

   - [
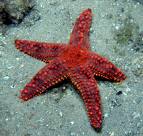
](http://www.mpa.nsw.gov.au/images/explore/psglmp-starfish.jpg)
   -
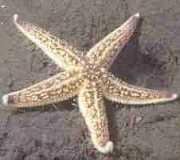

   - Don’t know
3. Which of the following pictures do you think is the New Zealand screw shell (*Maoriocolpus roseus*)?
   - [
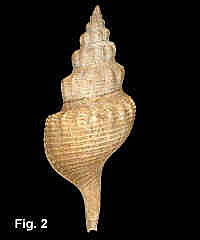
](http://seashellsofnsw.org.au/Buccininae/Images/0811-1.jpg)
   -
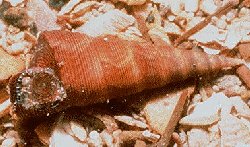

   -
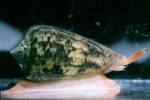

   - Don’t know
4. Which of the following pictures do you think is the European green crab (*Carcinus maenus*)?
   -
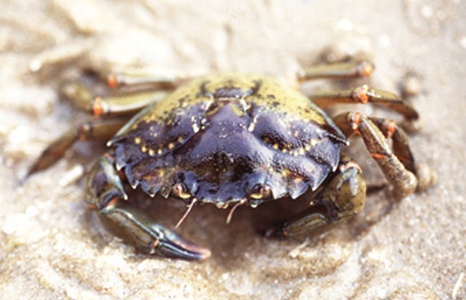

   - [
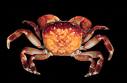
](http://images.google.com/imgres?imgurl=http://wdfw.wa.gov/fish/ans/identify/species_images/cyclograpsus_granulosus.jpg&imgrefurl=http://wdfw.wa.gov/fish/ans/identify/search.php?searchby=SpeciesClassification&search=Regulated&orderby=SpeciesFamily%20ASC&usg=__qWmINHUpIjNar1jnebE6q4SIsBQ=&h=293&w=450&sz=19&hl=en&start=1&um=1&tbnid=kzbJyct_5dB1UM:&tbnh=83&tbnw=127&prev=/images?q=cyclograpsus+granulosus&hl=en&rls=com.microsoft:*:IE-SearchBox&rlz=1I7GGIK_en&sa=N&um=1)
   -
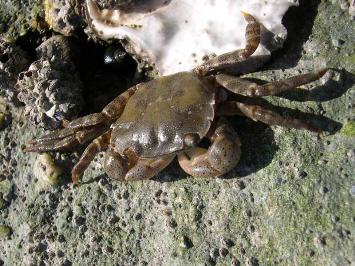

   - Don’t know
5. Have you seen any invasive species in this locality?
   - Yes
   - No
   - Unsure
6. If so which ones?
7. What did you do about it?
   - Removed it
   - Removed and report it
   - Reported the finding, only
   - Nothing
8. If you answered ‘nothing’ to Question 17 can you please explain why?
   - I was too busy to do something
   - I did not know where to report the finding
   - I thought it was already know to be there
   - I felt that it was not a big deal

**Please rate the following introduced species questions from strongly disagreeing to strongly agreeing (select only 1 choice per question).**

1. Introduced species increase biodiversity?
   - Strongly disagree
   - Disagree
   - Neutral
   - Agree
   - Strongly agree
2. Introduced species can lead to a reduction of native species?
   - Strongly disagree
   - Disagree
   - Neutral
   - Agree
   - Strongly agree
3. Introduced species out compete native species?
   - Strongly disagree
   - Disagree
   - Neutral
   - Agree
   - Strongly agree
4. Introduced species can reduce the aesthetic value of an area?
   - Strongly disagree
   - Disagree
   - Neutral
   - Agree
   - Strongly agree
5. Introduced species are health and safety risks?
   - Strongly disagree
   - Disagree
   - Neutral
   - Agree
   - Strongly agree
6. Introduced species can ruin the cultural value of an area?
   - Strongly disagree
   - Disagree
   - Neutral
   - Agree
   - Strongly agree
7. MPAs keep introduced species out of the MPA boundary
   - Strongly disagree
   - Disagree
   - Neutral
   - Agree
   - Strongly agree
8. Boats do not transport introduced species from one place to another
   - Strongly disagree
   - Disagree
   - Neutral
   - Agree
   - Strongly agree
9. Please consider and rate what level of impact (from highest to least) that you think introduced species have on YOUR marine activities, specifically?
   - Loss of biodiversity
     1. High impact
     2. Medium impact
     3. Low impact
     4. Least impact
   - Reduction in native species
     1. High impact
     2. Medium impact
     3. Low impact
     4. Least impact
   - Out competing native species
     1. High impact
     2. Medium impact
     3. Low impact
     4. Least impact
   - Loss of aesthetic value
     1. High impact
     2. Medium impact
     3. Low impact
     4. Least impact
   - Loss of cultural value
     1. High impact
     2. Medium impact
     3. Low impact
     4. Least impact
